# Antipsychotic Withdrawal Symptoms: A Systematic Review and Meta-Analysis

**DOI:** 10.3389/fpsyt.2020.569912

**Published:** 2020-09-29

**Authors:** Lasse Brandt, Tom Bschor, Jonathan Henssler, Martin Müller, Alkomiet Hasan, Andreas Heinz, Stefan Gutwinski

**Affiliations:** ^1^Department of Psychiatry and Psychotherapy, Charité Campus Mitte, Charité Universitätsmedizin Berlin, Corporate Member of Freie Universität Berlin, Humboldt-Universität zu Berlin, and Berlin Institute of Health, Berlin, Germany; ^2^Department of Psychiatry and Psychotherapy, Technical University of Dresden, Dresden, Germany; ^3^Department of Emergency Medicine, Inselspital, Bern University Hospital, University of Bern, Bern, Switzerland; ^4^Institute of Health Economics and Clinical Epidemiology, University Hospital of Cologne, Cologne, Germany; ^5^Department of Psychiatry and Psychotherapy, University Hospital Munich, Munich, Germany; ^6^Department of Psychiatry, Psychotherapy and Psychosomatics of the University Augsburg, Bezirkskrankenhaus Augsburg, Medical Faculty, University of Augsburg, Augsburg, Germany

**Keywords:** antipsychotics, withdrawal symptoms, discontinuation symptoms, systematic review, meta-analysis

## Abstract

**Objective:**

Avoiding withdrawal symptoms following antipsychotic discontinuation is an important factor when planning a safe therapy. We performed a systematic review and meta-analysis concerning occurrence of withdrawal symptoms after discontinuation of antipsychotics.

**Data Sources:**

We searched the databases CENTRAL, Pubmed, and EMBASE with no restriction to the beginning of the searched time period and until October 1, 2019 (PROSPERO registration no. CRD42019119148).

**Study Selection:**

Of the 18,043 screened studies, controlled and cohort trials that assessed withdrawal symptoms after discontinuation of oral antipsychotics were included in the random-effects model. Studies that did not implement placebo substitution were excluded from analyses. The primary outcome was the proportion of individuals with withdrawal symptoms after antipsychotic discontinuation. We compared a control group with continued antipsychotic treatment in the assessment of odds ratio and number needed to harm (NNH).

**Data Extraction:**

We followed guidelines by the Cochrane Collaboration, PRISMA, and MOOSE.

**Results:**

Five studies with a total of 261 individuals were included. The primary outcome, proportion of individuals with withdrawal symptoms after antipsychotic discontinuation, was 0.53 (95% CI, 0.37–0.70; *I^2^* = 82.98%, *P* < 0.01). An odds ratio of 7.97 (95% CI, 2.39–26.58; *I^2^* = 82.7%, *P* = 0.003) and NNH of 3 was calculated for the occurrence of withdrawal symptoms after antipsychotic discontinuation.

**Conclusion:**

Withdrawal symptoms appear to occur frequently after abrupt discontinuation of an oral antipsychotic. The lack of randomized controlled trials with low risk of bias on antipsychotic withdrawal symptoms highlights the need for further research.

## Introduction

Antipsychotics are primarily used in the treatment of psychotic disorders, which are in most cases associated with delusions and hallucinations (1). One of the most important indications are positive symptoms in schizophrenia (1). Furthermore, antipsychotics are used in a variety of other mental illnesses, such as bipolar affective disorder, psychotic depression, and agitation in neurodegenerative disorders.

Antipsychotic drugs are a heterogeneous group of compounds with a high variability of receptor affinities (2). The clinical effects of the individual compounds are diverse, since receptor affinity is an important factor for the efficacy, but especially for the side effect profile (3) and possibly for the withdrawal symptoms (4). The definition of a specific antipsychotic withdrawal syndrome is being debated (4, 5) and withdrawal symptoms may be associated with adverse events not previously complained of by the patient (6) such as cholinergic, dopaminergic, serotonergic, histaminergic, and adrenergic symptoms (2). Providing the appropriate discontinuation strategy for antipsychotic compounds is a complex task and depends on the side effect profile, pharmacodynamics and -kinetics of the compound, behavioral mechanisms as well as comorbidities and vulnerabilities of the patient (4, 7). However, clinical data is partly based on trials from prior to the 1980s (8). Recent research (4, 5) and schizophrenia guidelines (9) highlight the need for research in this field. To reduce the risk to develop long-term side effects, such as metabolic syndrome or tardive dyskinesia, different guidelines (e.g., by the National Institute for Health and Clinical Excellence in the United Kingdom (10)) recommend dose reduction or in certain cases antipsychotic withdrawal especially after the first psychotic episode.

Occurrence of withdrawal symptoms is not limited to psychopharmacological drugs (11) such as antipsychotics or antidepressants (12) or psychiatric patients (13) but have also been reported in other compounds such as beta-adrenergic receptor antagonists (14) or alpha-2 adrenergic agents (15) (used in the treatment of arterial hypertension) and can be expected following the abrupt discontinuation of many drugs (14). Individuals with antipsychotic withdrawal symptoms typically do not fulfil the addiction criteria as defined by the International Classification of Diseases (ICD-10 (11, 16). The main mechanism in the development of withdrawal symptoms is considered to be neuroadaptation to the predominantly antagonist effects of antipsychotics on different receptor systems and reactive homeostasis that is lost when dosing intake is stopped but the exact process remains to be elucidated (4, 5).

The primary research question (PICOS framework) of this systematic review and meta-analysis was: In patients treated with antipsychotics, what is the effect of antipsychotic discontinuation compared to no antipsychotic discontinuation or in uncontrolled designs on the occurrence of withdrawal symptoms?

### METHODS

The protocol for this systematic review and meta-analysis has *a priori* been published (PROSPERO registration no. CRD42019119148). We followed guidelines by the Cochrane Collaboration for conducting systematic reviews (17) and the Preferred Reporting Items for Systematic Reviews and Meta-Analyses (PRISMA) (18) and Meta-analysis of Observational Studies in Epidemiology (MOOSE) for extracting and assessing data (19).

### Search Strategy

The database search included CENTRAL, Pubmed, and EMBASE with no restriction to the beginning of the searched time period and until October 1, 2019. Title, abstracts, and key words were searched with terms for antipsychotic compounds and withdrawal or discontinuation: “([terms for specific antipsychotic compounds separated by OR] OR antipsychotic* OR neuroleptic*) AND (discontinu* OR withdraw*)”. We included terms for all antipsychotic compounds classified in the The Anatomical Therapeutic Chemical (ATC) classification system by the WHO Collaborating Centre for Drug Statistics Methodology (Supplement Section 1 – search entry) (20). A selective search of references in the included studies and other reviews was implemented to identify additional records. No language restrictions were included, and we acquired translations from a native speaker to test eligibility criteria of articles written in languages other than English. The literature search, study screening, and testing of eligibility criteria were carried out independently by two MDs (LB, SG) with Endnote, version X8.2 (Clarivate Analytics). We contacted authors of the included studies in case of missing information provided contact information could be retrieved.

### Eligibility Criteria

Articles were included when they fulfilled the following criteria. First, occurrence of antipsychotic withdrawal symptoms was assessed quantitatively. Second, the study was designed as a controlled or cohort trial. Third, the group under investigation for withdrawal symptoms received a placebo substitute after antipsychotic discontinuation. Fourth, the reported observation period covered at least 7 days after antipsychotic discontinuation (the observation period refers to the total duration that an individual was clinically observed for after antipsychotic discontinuation).

All types of discontinuation (e.g., abrupt or stepwise) and all study populations (e.g., individuals with psychiatric or non-psychiatric illnesses) were included. Regarding the antipsychotic treatment before discontinuation, the inclusion of studies was not restricted to a specific duration of antipsychotic treatment. Studies were excluded if there was no information on the exact number of individuals with withdrawal symptoms. Studies were excluded if they did not report daily clinical assessments of the individual (i.e., clinical assessment interval) because short lasting withdrawal symptoms previously reported in antipsychotics (4) and other psychopharmacological compounds such as antidepressants (12) otherwise may be missed. Studies with assessments of only single symptoms such as a sleep disorder were excluded.

### Study Selection, Data Collection, and Data Extraction

Study selection and classification and coding of data into an predefined Excel spreadsheet (Microsoft Excel for Mac, version 16.12, Microsoft Corporation) following the recommendations by the Cochrane Collaboration handbook (17) was carried out independently by two MDs (LB, SG). Discrepancies were resolved by consensus with additional review authors (JH, MM).

### Risk of Bias

We assessed the risk of bias according to the Cochrane Collaboration’s tool (21): Studies were assessed as holding a “low”, “unclear”, or “high” risk of bias in the six domains of selection bias, performance bias, detection bias, attrition bias, reporting bias, and other bias. Risk of bias was assessed independently by two MDs (LB, SG). Discrepancies were resolved by consensus with additional review authors (JH, MM).

### Data Synthesis

The primary outcome measure was withdrawal symptoms after antipsychotic discontinuation. A random effects meta-analysis of proportions (17) was carried out for the primary outcome of the main quantitative analysis and showed the weighted mean of the proportion of individuals with withdrawal symptoms after antipsychotic discontinuation and placebo substitution accompanied by its 95% CI. In the sensitivity analysis, only studies with an allocation of individuals to a target group that received a placebo substitute after antipsychotic discontinuation or a control group that continued antipsychotic treatment were included. The outcome of the sensitivity analysis was the pooled odds ratio (OR) for individuals who discontinued the antipsychotic treatment compared with individuals who continued the antipsychotic treatment. Number needed to harm (NNH) was calculated for individuals who discontinued the antipsychotic treatment compared with individuals who continued the antipsychotic treatment. Heterogeneity among studies was assessed with *I^2^* statistics. We intended to assess publication bias by funnel plots and Egger’s test as recommended by Sterne et al (22). All statistical analyses were performed with Stata, version 13.1 (StataCorp LLC). A 2-sided *P <*0.05 was considered statistically significant. Analyses were not corrected for multiple testing.

## Results

After removal of duplicates, 18,043 studies were retrieved through the literature search and screened (**Supplement Figure 1**). 7 of these screened studies were retrieved through manual searches. Studies were screened and 119 full-text manuscripts were assessed for eligibility. We excluded a total of 114 studies because of the following reasons: withdrawal symptoms were not assessed in 85 studies, 11 studies were case reports, no placebo substitute was implemented in nine studies, only single symptoms such as sleep disorders were assessed in five studies, the clinical assessment interval was not daily in two studies, the observation period was less than 7 days in one study, and no exact number of individuals was reported in one study. Five articles, published between 1959 and 1976, met the inclusion criteria for the quantitative analysis (**Table 1**).

**Table 1:** Included studies on the occurrence of withdrawal symptoms after abrupt antipsychotic discontinuation.

Study (publication year)	Diagnosis	Study design	Age and sex	Discontinued antipsychotic medication (dose per day, if specified)	Other medication discontinued	Observation period after antipsychotic discontinuation	Risk of bias according to Cochrane Collaboration’s tool for assessing risk of bias	Individuals with withdrawal symptoms in % (N)
Battegay (1966) (23)	Schizophrenia, “oligophrenia”, or “organic brain damage”	Control: No Rand.: N. a. Placebo: Yes Blinded: No	44% <50 years and 56% >50 years, 57% females^F^	Derivates of phenotiazine, thiaxanthene, butyrophenone, or piperazinyl^F^	“antiparkinson agents” biperidene or ethybenzatropin in all individuals of this subgroup “antidepressants” in 6% and diazepam in 4% of all individuals of the study^F^	7 days	High	Target group: 86% (12/14)
					“antidepressants” in 6% and diazepam in 4% of all individuals in the study^F^	7 days	High	Target group: 59% (24/41)
Brooks (1959) (24)	Schizophrenia^E^	Control: No Rand.: N. a. Placebo: Yes Blinded: No	19–53 years, 100% females	Chlorpromazine (150–600mg) and/or reserpine (1.5–4mg)	trihexiphenidyl, procyclidine hydrochloride, or benztropine methanesulfonate in “most” individuals intended to “relief extrapyramidal symptoms”	10 days	High	Target group: 61% (17/28)^A^
Degkwitz et al. (1970) (25)	Schizophrenia^E^	Control: Yes Rand.: N. s. Placebo: Yes^C^ Blinded: Yes^C^	20–79 years, 62.5% females	Antipsychotic medication not specified (mean 388 chlorpromazine units per day in the target group)	None	5-7 months	High	Target group: 36% (19/53)^B^ Control group: 11% (4/35)^B^
Lacoursiere et al. (1976) (8)	Schizophrenia^E^	Control: Yes Rand.: N. s. Placebo: Yes Blinded: Yes	Mean 37 years, 29% females	Chlorpromazine and/or other unspecified antipsychotics (mean 800 chlorpromazine units per day)	Benztropine mesylate as “antiparkinson agent” in all individuals	4 weeks	High	Target group 25% (4/16)
					None	4 weeks	High	Target group 38% (10/26) Control group: 12.5% (1/8)
Melnyk et al. (1966) (26)	Schizophrenia^D^	Control: Yes Rand.: Yes Placebo: Yes Blinded: Yes	Age n. s., 50% females (target: 65% females; control: 35% females)	Thioridazine or chlorpromazine (100-600mg)	None	6 weeks	High	Target group: 75% (15/20) Control group: 10% (2/20)

N. s., Not specified; N. a., Not applicable; Control, Control group, which continued antipsychotic treatment; Rand., randomized allocation (to a target group, which discontinued antipsychotic treatment, or a control group, which continued antipsychotic treatment); Placebo, the target group received a placebo substitute after antipsychotic discontinuation; Blinded, the trial was double-blinded and both participating individuals and investigators blinded. ^A^Brooks focused on withdrawal symptoms that were of at least moderate degree, i.e., “marked and distressing”, which he detected in 17 individuals. Brooks did not include 9 other individuals with “mild reactions” also excluded from our analysis. ^B^New vegetative symptoms and hyperkinesia. ^C^Placebos biased by not completely identical taste in the study by Degkwitz et al. ^D^Individuals with “paranoid, undifferentiated, catatonic, or hebephrenic schizophrenia” and one individual with “acute schizophrenic reaction”. ^E^“Chronic schizophrenia”. ^F^Battegay reported age and sex distribution, antipsychotic medication, and other medication for the entire study (n = 81) including a subgroup without placebo substitution (n = 26/81), which was excluded from this meta-analysis.

Three out of the five eligible studies (studies by Degkwitz et al. (25), Lacoursiere et al. (8), and Melnyk et al. (26); **Table 1**) in the quantitative analysis had implemented a double-blinded design, setting the participants to placebo (i.e., target group) or to continuation of antipsychotic treatment (i.e., control group). The other two out of the five eligible studies in the quantitative analysis (i.e., studies by Battegay (23)). and Brooks (24); **Table 1**) were cohort studies setting the participants to placebo (i.e., target group) and no control group that continued the antipsychotic. Individuals in the target group of all included studies had discontinued antipsychotics abruptly. No individuals in the included studies discontinued antipsychotics stepwise (also known as tapered discontinuation). Study populations primarily focused on individuals with schizophrenia but diagnostic procedures (e.g., ICD or DSM) were not specified (**Table 1**). Individuals were assessed clinically daily in all studies. Lacoursiere et al. did not report the daily assessments in the publication but confirmed it through email correspondence with the authors on January 2, 2019. The observation periods showed a high variability from 7 days to 7 months. The discontinued antipsychotic medication comprised different oral dopamine (D2) receptor antagonists (i.e., also known as “first-generation neuroleptics/antipsychotics”) in monotherapy or combination therapy. In the study by Battegay, individuals also stopped other co-medication (“antidepressants” in 6% of individuals and diazepam in 4% of individuals). Battegay, Brooks, and Lacoursiere et al. included cohorts that also discontinued medication intended to relief “extrapyramidal” or “parkinson” symptoms (i.e., biperidene, ethybenzatropin, benztropine methanesulfonate, trihexiphenidyl, or procyclidine hydrochloride). Lacoursiere et al. reported separate results for cohorts with additional “antiparkinson agents”. Cohorts with discontinued other medication than antipsychotics were not included in the sensitivity analysis. None of the included studies reported a specific standardized clinical assessment procedure for withdrawal symptoms. Brooks focused on withdrawal symptoms that were of at least moderate degree, i.e., “marked and distressing”, which he detected in 17 individuals. Brooks did not include nine other individuals with “mild reactions”. These “mild reactions” were not specified and also excluded from our analysis. All studies showed a “high” risk of bias according to Cochrane Collaboration’s tool for assessing risk of bias (**Table 1**) (21).

Reported symptoms included nausea and vomiting, abdominal pain, diarrhea, headache, tachycardia, vertigo, increased perspiration, dry mucous membranes, myalgia, restlessness, anxiety, tension, insomnia, and hyperkinesia (**Table 2**). In addition, Lacoursiere et al. described “isolated reports of numbness, nightmares, rhinorrhea, and bad taste”. Battegay (23) reported a significantly higher rates of withdrawal symptoms in women and the age group above 50 years after discontinuation of different antipsychotics (23).

**Table 2:** Clinical features of abrupt oral antipsychotic withdrawal.

Symptoms	Nausea and vomiting (8, 24, 26)
	Abdominal pain (26)
	Diarrhea (25)
	Headache (8, 23, 26)
	Tachycardia (23, 25)
	Vertigo (8, 23)
	Increased perspiration (23, 25, 26)
	Dry mucous membranes (25)
	Myalgia (8, 26)
	Restlessness (8, 24–26)
	Anxiety (24)
	Tension (24)
	Insomnia (8, 23–25)
	Hyperkinesia (23, 25)
Time of onset	Within 4 weeks after discontinuation (8, 23–26)
Duration	1–4 weeks (hyperkinesia may last months) (8, 23–26)
Proportion of individuals with withdrawal symptoms	0.53 (95% CI, 0.37–0.70)^A^ (8, 23–26)
Odds ratio (OR)	7.97 (95% CI, 2.39–26.58; *I^2^* = 82.7%, *P* = 0.003)^B,^ (8, 25–26)
Number needed to harm (NNH)	3^B^ (8, 25–26)

^A^Based on main analysis of five trials that abruptly substituted oral antipsychotics with placebo (**Figure 1**), the sensitivity analysis showed similar results and a weighted mean of 0.49 (95% CI, 0.26–0.73; **Figure 2**). ^B^analysis was restricted to the three studies in the sensitivity analysis with an allocation to a target group that received a placebo substitute after abrupt antipsychotic discontinuation or a control group that continued antipsychotic treatment with a double-blinded design (**Figure 3**).

### Main Analysis

**Figure 1:**
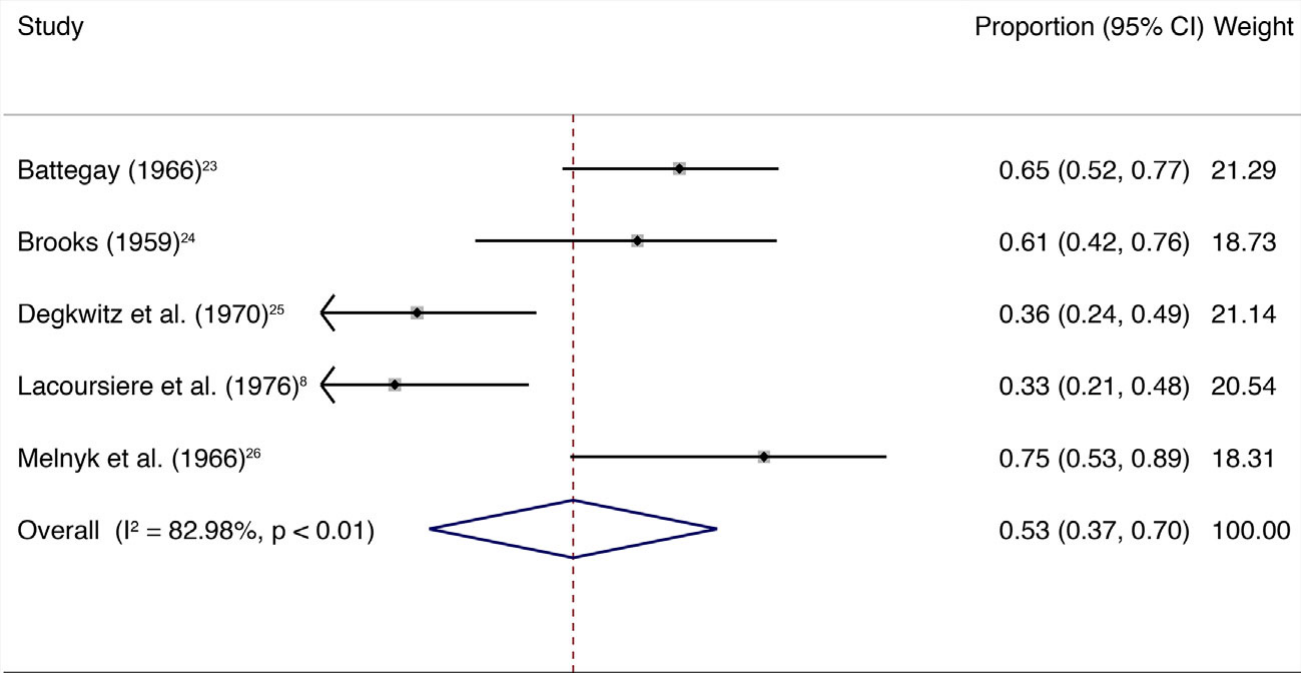
The square data markers indicate the proportion of individuals with withdrawal symptoms in each study, with sizes reflecting the study’s statistical weight using random-effects meta-analysis of proportions. The horizontal lines indicate 95% CIs. The blue diamond data marker represents the overall proportion and 95% CI. The vertical dashed line shows the summary effect estimate.

The main quantitative analysis included five studies with a total of 261 individuals (**Figure 1**). A weighted mean of 0.53 (95% CI, 0.37–0.70) of individuals showed withdrawal symptoms after abrupt discontinuation of oral antipsychotic treatment and placebo substitution. Heterogeneity among the included trials was high (*I^2^* = 82.98%, *P* < 0.01; **Figure 1**). Numbers of included studies were too low (n = 5) to adequately assess publication bias by funnel plot inspection or more advanced regression-based assessments.

### Sensitivity Analyses

**Figure 2:**
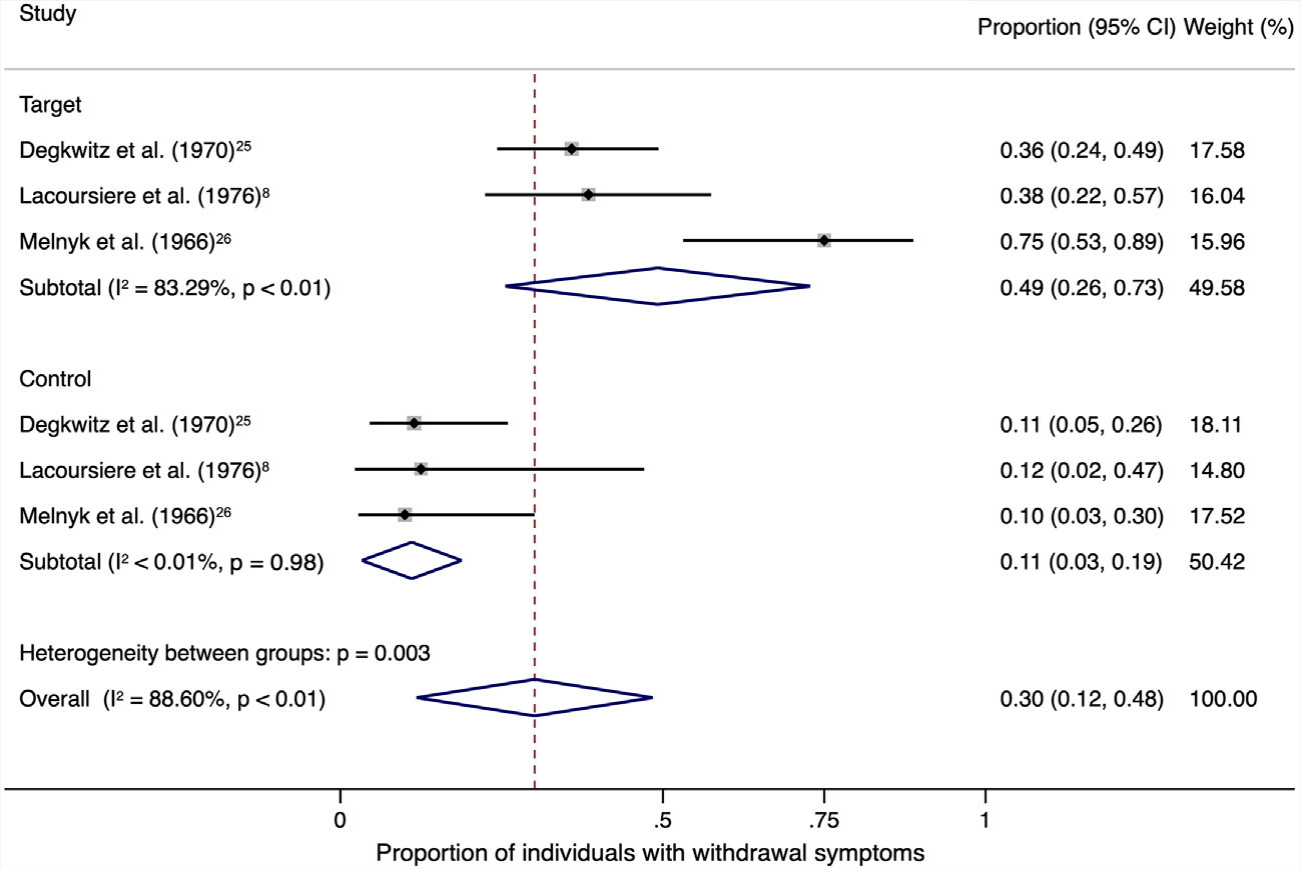
The square data markers indicate the proportion of individuals with withdrawal symptoms in each study, with sizes reflecting the statistical weight of the study using random-effects meta-analysis of proportions. The horizontal lines indicate 95% CIs. The blue diamond data marker represents the subtotal and overall proportion and 95% CI. The vertical dashed line shows the overall effect estimate for both target and control together.

The sensitivity analysis was limited to three studies with a target and a control group and total of 162 individuals (**Figure 2**). A weighted mean of 0.49 (95% CI, 0.26–0.73) of individuals showed withdrawal symptoms after abrupt discontinuation of oral antipsychotic treatment and placebo substitution in the target group. In comparison, the control group only showed a weighted mean of 0.11 (95% CI, 0.03–0.19) after continuation of oral antipsychotic treatment.

Heterogeneity among the included trials was high in the target group (*I^2^* = 83.29%, *P* < 0.01) and low in the control group (*I^2^* < 0.01%, *P* = 0.98).

**Figure 3:**
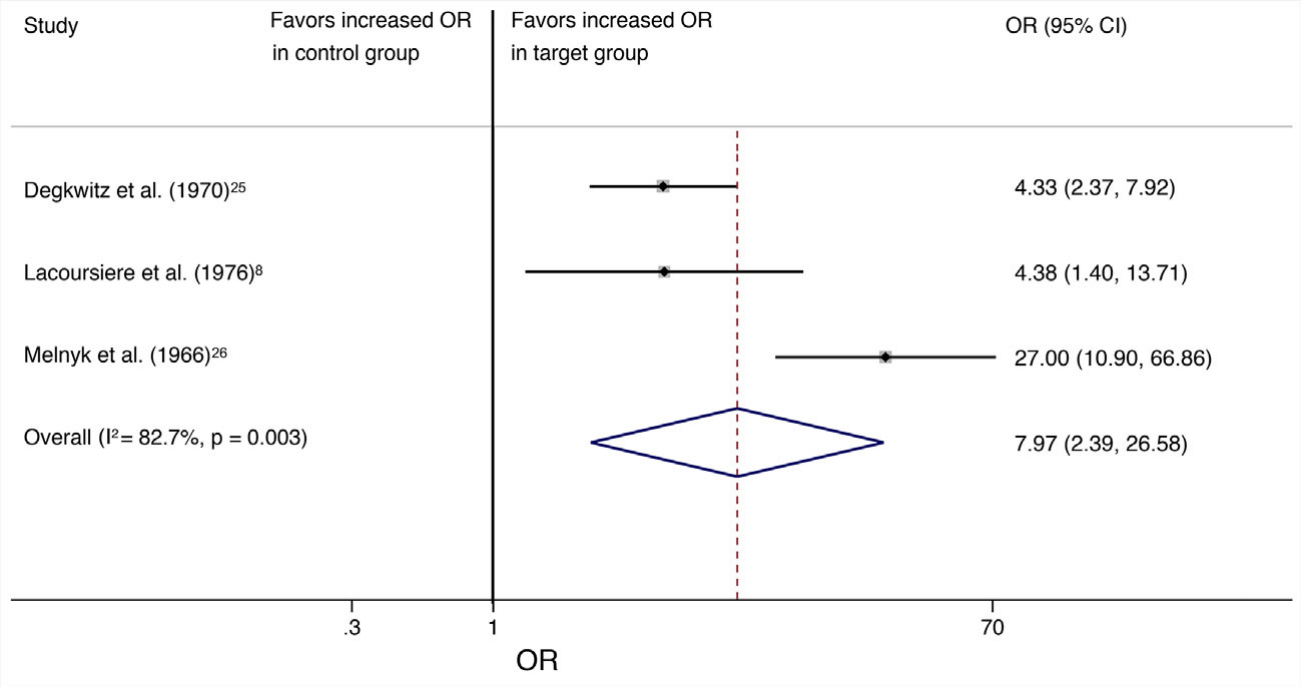
The square data markers indicate the proportion of individuals with withdrawal symptoms in each study, with sizes reflecting the statistical weight of the study using random-effects meta-analysis. The horizontal lines indicate 95% CIs. The blue diamond data marker represents the overall proportion and 95% CI. The vertical dashed line shows the overall effect estimate, and the continuous line represents the line of no effect (OR = 1).

Odds ratio resulted in 7.97 (95% CI, 2.39–26.58; *I^2^* = 82.7%, *P* = 0.003) and number needed to harm (NNH) was 3 when analyses were restricted to the three studies with allocation to a target group that received a placebo substitute after abrupt antipsychotic discontinuation and a control group that continued antipsychotic treatment (**Figure 3**).

## Discussion

To our knowledge, this is the first systematic review and meta-analysis on the occurrence of withdrawal symptoms after antipsychotic discontinuation. A weighted average of 53% individuals showed withdrawal symptoms after abrupt antipsychotic discontinuation and placebo substitution in this meta-analysis.

The studies in the main quantitative analysis were characterized by high risk of bias due to methodological heterogeneity, discontinuation of different antipsychotic and non-antipsychotic compounds, and considerable missing information. The proportion of individuals with withdrawal symptoms in the target groups showed a high variability, presumably because of the heterogeneity of study designs and in particular the use of different antipsychotics as monotherapy or as combination therapy. The studies by Battegay (23) and Brooks (24) included no placebo arm with continued antipsychotic treatment and were excluded in the sensitivity analysis. All cohorts with discontinuation of other medication than antipsychotics were also excluded from the sensitivity analysis. The sensitivity analyses supported the results of the main analysis and showed a weighted average of 49% in the target group versus 11% in the control group, an odds ratio of 7.97, and NNH of 3. In this sensitivity analysis, the proportion of individuals with symptoms in the control group (continued intake of antipsychotics) showed a comparatively low variability of 10–12% across studies. The effect sizes should still be considered as guiding estimates and not exact values due to high risk of bias.

Withdrawal symptoms after abrupt antipsychotic discontinuation in the included studies were heterogenous and affected multiple organ systems (**Table 2**). Symptoms can be very related (e.g., agitation and insomnia) or unlike (e.g., vomiting) to the beginning of a psychotic relapse. In addition, Degkwitz et al. reported that all individuals with vegetative withdrawal symptoms and/or hyperkinesia also worsened in psychopathological scores (25). These findings demonstrate that attributing a particular symptom to either withdrawal or the beginning of a psychotic relapse can be a complex task (4, 5, 27) and emphasizes the need for further psychopathological assessment and a close monitoring of the development of the symptoms.

The studies in our review (8, 23–26) reported that most withdrawal symptoms started within 4 weeks after abrupt antipsychotic discontinuation and subsided after up to 4 weeks even though certain symptoms such as hyperkinesia may last for months (23).

A randomized controlled trial (RCT) by Tollefson et al. from 1999 seemed to be in contrast to the findings from this meta-analysis (28): they did not detect increased rates of symptoms such as nausea, vomiting, diarrhea, or headache in the group that received placebo after discontinuation of clozapine 300 mg per day. The contrast between the RCT and the included studies in this meta-analysis may be due to the fact that placebo was administered for three to five days and potential withdrawal symptoms could only be observed in this shorter period of time. Withdrawal symptoms with a later onset were not investigated in the study by Tollefson et al. In comparison, Shiovitz et al. assessed individuals for seven days after discontinuation of clozapine 200 mg per day without placebo substitution and detected withdrawal symptoms in 61% of the 28 individuals (29).

Only few data regarding risk factors for the occurrence of withdrawal symptoms have been reported (4). Battegay observed that the occurrence of withdrawal symptoms after discontinuation of different antipsychotics was significantly higher in women and the age group above 50 years (23). Degkwitz et al. also reported a higher rate of withdrawal symptoms in women (30) and Azermai et al. detected a high rate of individuals with withdrawal symptoms (72%) in a geriatric population (31). These findings raise the question, if the female and geriatric population are particularly vulnerable for the development of withdrawal symptoms after antipsychotic discontinuation. No significant effect of dosage on the type and severity of withdrawal symptoms was reported in two other studies (last medication: reserpine 1.5–4 mg per day and/or chlorpromazine 150–600 mg per day) (8, 24). However, it is not known whether dose effects would be detected if lower dosages would be included in the analyses (8).

Several smaller (n ≤ 30 participants per study) studies reported specific aspects of sleep disorders after antipsychotic withdrawal such as mean decreased REM sleep (32–34), reduced total sleep duration (34), decreased sleep efficiency (35), or decreased sleep continuity (33). In these studies, the sleep disorders were detected during the first days to weeks following withdrawal from first generation antipsychotics (e.g., haloperidol or mesoridazine) or second generation antipsychotics (e.g., risperidone or olanzapine) (32–35).

Abrupt withdrawal of haloperidol was associated with a significant increase in dyskinesia beginning in the second week post-withdrawal in a study with fifteen participants with schizophrenia (36). A case series (37) and case report (38) indicated occurrence of dyskinesia and dystonia after abrupt withdrawal of clozapine. In another study with 34 children, 6 to 12 years of age and diagnosed with schizophrenia, 41% of the children showed involuntary movements and ataxia after withdrawal of different antipsychotics (i.e., fluphenazine, haloperidol, thioridazine, trifluoperazine, and thiothixene) (39). In half of the children with involuntary movements and ataxia, symptoms did not disappear spontaneously within 2 to 5 weeks after withdrawal but only after antipsychotic treatment was started again (39).

The included studies in this systematic review and meta-analysis did not implement stepwise discontinuation but Greenberg and Roth compared abrupt discontinuation (n = 21 persons) with a stepwise and slow discontinuation over the duration of 336 days (n = 21 persons) of chlorpromazine (initial average dose of 510 mg per day) (40). They reported that the “majority” of individuals after abrupt discontinuation suffered from withdrawal symptoms compared to none of the individuals after stepwise reduction. A recent study by Emsley et al. from 2018 showed no significant difference in blood pressure and heart rate in individuals after discontinuation of long acting paliperidone palmitate once monthly injections compared to individuals with continued treatment (41). Based on these limited findings, it could be hypothesized that the long acting kinetic properties with a very slow decrease of antipsychotic dosage over weeks may reduce the occurrence of withdrawal symptoms similar to the stepwise reduction in the study by Greenberg and Roth. In a meta-analysis by Leucht et al. (42), the authors reported that the increased occurrence of dyskinesia was probably related to abrupt antipsychotic withdrawal, but assessment of the disorder by appropriate criteria was lacking (42). Furthermore, specific tapering strategies to reduce the risk of withdrawal symptoms were recently recommended for antidepressants: For example, Horowitz and Taylor (43) proposed a slow and hyperbolical taper strategy to mitigate SSRI withdrawal symptoms (43).

It has been suggested that even dose reductions without complete discontinuation of antipsychotics may cause withdrawal symptoms (5). More research is needed to investigate the risk of withdrawal symptoms prior to complete discontinuation such as during different tapering procedures or during dose reductions without complete discontinuation.

The findings from this meta-analysis indicate that withdrawal symptoms after abrupt antipsychotic discontinuation occur frequently. This finding is of importance when planning a safe therapy and implicates that the risk of withdrawal symptoms after antipsychotic discontinuation requires consideration in clinical practice and health policy. Future research may also assess the effect antipsychotic withdrawal symptoms on other parameters such as psychosocial functioning and quality of life. Current schizophrenia guidelines (e.g., by the National Institute for Health and Clinical Excellence in the United Kingdom (10) or German Association for Psychiatry, Psychotherapy and Psychosomatics (9)) provide information on how to prepare and approach discontinuation of antipsychotics (e.g., tapering strategies).

This finding also has implications for the design and interpretation of clinical trials with antipsychotics. For example, a washout phase at the beginning of a clinical trial could possibly lead to an increase of adverse events unrelated to the trial medication or placebo if the duration of the washout phase is shorter than the duration of withdrawal symptoms from the individual prestudy antipsychotics. Little is known about the effect of withdrawal symptoms on the occurrence of adverse events in clinical trials with antipsychotics and more research is needed to elucidate this potentially critical effect.

Future research may address effects of antipsychotic withdrawal in different psychiatric diagnoses and take into account whether effects are dependent on dose or duration of antipsychotic treatment and pharmacological characteristics such as elimination half-life or anticholinergic properties.

### Limitations

This systematic review and meta-analysis confirmed the initial assumption that withdrawal symptoms occur frequently after abrupt antipsychotic discontinuation. The representativeness and validity of our findings are, however, limited by the following aspects:

The included studies are few, with small number of subjects, not recent, methodologically heterogenous, and characterized by a high risk of bias. So-called second-generation antipsychotics, which are considered first-line treatment and differ in their receptor profiles, were not included. The lack of recent discontinuation studies may be related to ethical considerations, as discontinuation of antipsychotics increases the risk of relapse. Therefore, the existing data are largely from studies published before 1980. Moreover, it remains elusive whether individuals following the first exposure to an antipsychotic are at different risk to develop withdrawal symptoms than patients following a chronic exposure to an antipsychotic. Large high quality randomized controlled trials with a focus on discontinuation with antipsychotics are needed. The proportion of individuals with withdrawal symptoms may further differ according to characteristics of individuals such as age, sex, and specific types of antipsychotic drugs (23). Drawing conclusions from this meta-analysis should consider these limitations and results may thus be used as guiding values only.

## Conclusion

In this systematic review and meta-analysis of five studies, the proportion of individuals with withdrawal symptoms after abrupt oral antipsychotic discontinuation was increased. In studies with a control group that continued antipsychotic treatment, only a proportion of 0.11 of individuals in the control group showed withdrawal symptoms compared to a proportion of 0.49 of individuals after abrupt antipsychotic discontinuation (odds ratio 7.97 and NNH 3); available information was limited to studies with so-called first-generation antipsychotics and a high risk of bias. These findings suggest that withdrawal symptoms may occur frequently after abrupt discontinuation of oral antipsychotics but the lack of randomized controlled trials with low risk of bias highlights the need for further research.

## Data Availability Statement

The original contributions presented in the study are included in the article/**Supplementary Material**, further inquiries can be directed to the corresponding author and first author.

## Author Contributions

Concept and design: LB, JH, AHe, and SG. Acquisition, analysis, or interpretation of data: All authors. Drafting of the manuscript: LB, AHa, AHe, and SG. Critical revision of the manuscript for important intellectual content: All authors. Statistical analysis: LB, JH, MM, and SG. Administrative, technical, or material support: LB, TB, MM, and SG. Supervision: TB, AHe, and AHa.

## Funding

We acknowledge support from the German Research Foundation (DFG) and the Open Access Publication Fund of Charité – Universitätsmedizin Berlin.

## Conflict of Interest

AHa is Editor of the World Federation of Societies of Biological Psychiatry (WFSBP) guideline schizophrenia and co-editor of the German S3-guideline schizophrenia. He was member of an advisory board of Janssen, Lundbeck, and Otsuka and received payed speakership for scientific talks from these companies.

The remaining authors declare that the research was conducted in the absence of any commercial or financial relationships that could be construed as a potential conflict of interest.

## Supplementary Material

Supplementary Figure 1 PRISMA flowchart.

Supplementary Data Sheet 1 Database search entry.
