# Supplementary figures and images for: Antipsychotic Withdrawal Symptoms: A Systematic Review and Meta-Analysis
Source: Front Psychiatry. 2020 Sep 29;11:569912. doi: 10.3389/fpsyt.2020.569912 (PMC7552943; doi:10.3389/fpsyt.2020.569912)

**Supplementary Figure 1. PRISMA flowchart.**

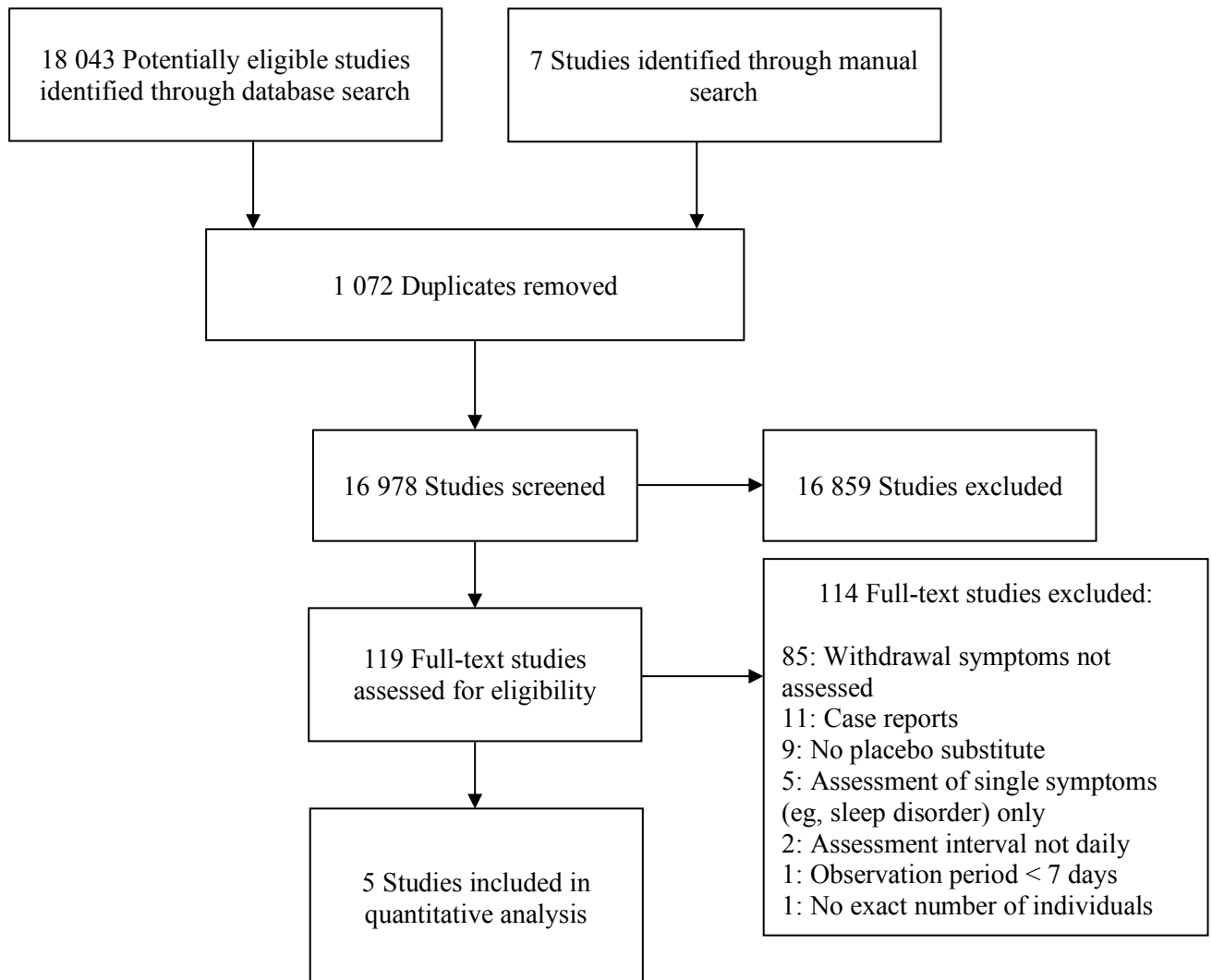

Supplement: Supplementary Figure 1 — PRISMA flowchart. [file Image_1.pdf]
