# Supplementary material for: Antipsychotic Withdrawal Symptoms: A Systematic Review and Meta-Analysis
Source: Front Psychiatry. 2020 Sep 29;11:569912. doi: 10.3389/fpsyt.2020.569912 (PMC7552943; doi:10.3389/fpsyt.2020.569912)
Supplement: Supplementary Data Sheet 1 — Database search entry. [file DataSheet_1.pdf]

Supplement Section 1: ***Database search entry***

All antipsychotic compounds classified in the WHO ATC classification system[20] were included in the search terms:

((Acepromazine OR Acetophenazine OR Benperidol OR Bromperidol OR Butaperazine OR Carfenazine OR Chlorproethazine OR Chlorpromazine OR Chlorprothixene OR Clopenthixol OR Cyamemazine OR Dixyrazine OR Droperidol OR Fluanisone OR Flupentixol OR Fluphenazine OR Fluspirilene OR Haloperidol OR Levomepromazine OR Lenperone OR Loxapine OR Mesoridazine OR Metitepine OR Molindone OR Moperone OR Oxypertine OR Oxyprotepine OR Penfluridol OR Perazine OR Periciazine OR Perphenazine OR Pimozide OR Pipamperone OR Piperacetazine OR Pipotiazine OR Prochlorperazine OR Promazine OR Prothipendyl OR Spiperone OR Sulforidazine OR Thiopropazate OR Thioproperazine OR Thioridazine OR Thiothixene OR Timiperone OR Trifluoperazine OR Trifluperidol OR Triflupromazine OR Zuclopenthixol

OR

Amoxapine OR Amisulpride OR Aripiprazole OR Asenapine OR Blonanserin OR Brexpiprazole OR Cariprazine OR Carpipramine OR Clocapramine OR Clorotepine OR Clotiapine OR Clozapine OR Iloperidone OR Levosulpiride OR Lurasidone OR Melperone OR Mosapramine OR Nemonapride OR Olanzapine OR Paliperidone OR Perospirone OR Quetiapine OR Remoxipride OR Reserpine OR Risperidone OR Sertindole OR Sulpiride OR Sultopride OR Tiapride OR Veralipride OR Ziprasidone OR Zotepine

OR

antipsychotic* OR neuroleptic*)

AND

(discontinu* OR withdraw*)).

References for supplement

1. WHO Collaborating Centre for Drug Statistics Methodology. The Anatomical Therapeutic Chemical (ATC) classification system. https://www.whocc.no.
